# Pharmacokinetics and ex vivo anti‐inflammatory effects of oral misoprostol in horses

**DOI:** 10.1111/evj.13024

**Published:** 2018-10-23

**Authors:** E. M. Martin, J. M. Schirmer, S. L. Jones, J. L. Davis

**Affiliations:** ^1^ North Carolina State University College of Veterinary Medicine Raleigh North Carolina USA; ^2^ VA‐MD College of Veterinary Medicine Blacksburg Virginia USA

**Keywords:** horse, leucocyte, E prostanoid receptor agonist, pharmacokinetics, inflammation, tumour necrosis factor‐α

## Abstract

**Background:**

Misoprostol is an E prostanoid (EP) 2, 3 and 4 receptor agonist that is anecdotally used to treat and prevent NSAID‐induced GI injury in horses. Misoprostol elicits anti‐inflammatory effects in vivo in men and rodents, and inhibits TNFα production in equine leucocytes in vitro.

**Objective:**

Define the pharmacokinetic parameters of oral misoprostol in horses, and determine the inhibitory effect of oral misoprostol administration on equine leucocyte TNFα production in an ex vivo inflammation model.

**Study design:**

Pharmacokinetic study, ex vivo experimental study.

**Methods:**

Six healthy adult horses of mixed breeds were used. In phase one, horses were given 5 μg/kg misoprostol orally, and blood was collected at predetermined times for determination of misoprostol free acid (MFA) by UHPLC‐MS/MS. Pharmacokinetic parameters were calculated. In phase two, horses were dosed as in phase one, and blood was collected at T0, 0.5, 1 and 4 h following misoprostol administration for leucocyte isolation. Leucocytes were stimulated with 100 ng/mL LPS, and TNFα mRNA concentrations were determined via quantitative real‐time PCR.

**Results:**

About 5 μg/kg oral misoprostol produced a rapid time to maximum concentration (T_max_) of 23.4 ± 2.4 min, with a maximum concentration (C_max_) of 0.29 ± 0.07 ng/mL and area under the curve (AUC_0−∞_) of 0.4 ± 0.12 h ng/mL. LPS stimulation of equine leucocytes ex vivo significantly increased TNFα mRNA concentrations, and there was no significant effect of misoprostol even at the T_max_.

**Main limitations:**

Only a single dose was used, and sample size was small.

**Conclusions:**

Misoprostol is rapidly absorbed following oral administration in horses, and a single 5 μg/kg dose had no significant inhibitory effect on ex vivo LPS‐stimulated TNFα mRNA production in leucocytes. Further studies analysing different dosing strategies, including repeat administration or combination with other anti‐inflammatory drugs, are warranted.

## Introduction

Tumour necrosis factor α (TNFα) is a potent pro‐inflammatory cytokine that plays an important role in equine diseases such as mild to moderate equine asthma, systemic inflammatory response syndrome (SIRS) and laminitis 1, 2, 3. In particular, TNFα induces multiple leucocyte effector functions that elicit extensive tissue damage in disease states including sepsis, equine laminitis and equine asthma 4, 5, 6. Current anti‐inflammatory regimens used in equine medicine rely heavily on nonsteroidal anti‐inflammatory drugs (NSAIDs), which are associated with gastrointestinal (GI) effects, most notably in critically ill horses 7. These adverse effects are thought to be due to inhibition of gastroprotective prostaglandins 7, 8. Studies have demonstrated that addition of the PGE_1_ analogue, misoprostol, aids in recovery of NSAID‐injured equine jejunum in in vitro models of mucosal damage 9 and increases gastric pH in the equine stomach 10. While in vivo efficacy research is lacking, misoprostol is used clinically to treat and prevent NSAID‐induced gastroenteropathies. In addition to this role, misoprostol has been shown to elicit anti‐inflammatory effects on human leucocytes through an agonistic effect on the E‐type prostaglandin receptor, EP2 11, 12. EP2 agonists increase intracellular production of cyclic AMP (cAMP) 13, which dampens tissue‐damaging leucocyte effector functions including chemotaxis 14, adhesion and reactive oxygen species production 15, 16. Recent work has demonstrated that misoprostol at concentrations greater than 1 μmol/L inhibits multiple equine leucocyte functions in vitro including LPS‐stimulated TNFα production 17. Additionally, oral misoprostol at a dose of 5 μg/kg twice daily has been shown to aid in healing of equine gastric glandular disease (EGGD) lesions in vivo 18, the pathology of which is associated with inflammation of the gastric mucosa 19. While this suggests the possibility of local anti‐inflammatory effects within the gastrointestinal tract, the systemic anti‐inflammatory therapeutic potential of oral administration of misoprostol in horses has not yet been evaluated.

Despite widespread anecdotal clinical use, the pharmacokinetics of misoprostol has not yet been evaluated in horses. Additionally, the effects of oral misoprostol administration on equine leucocyte TNFα production have not yet been evaluated. Thus, the goal of this research was to define the pharmacokinetics of 5 μg/kg misoprostol administered orally in horses, as well as the effects of oral misoprostol on TNFα cytokine production in equine leucocytes.

## Materials and methods

This study was conducted in two phases: the first was a misoprostol free acid pharmacokinetic study, and the second was an ex vivo experimental study determining the effect of oral misoprostol on TNFα mRNA concentrations. Horses received identical misoprostol dosing in each phase (see methods below), and the second phase was conducted approximately 3 months following the phase one pharmacokinetic study to allow ample time for misoprostol washout between experiments. The same six horses were used for both phases of the study.

### Equine subjects

Six adult, castrated male horses of various breeds were used. Horses weighed between 470 and 650 kg, and ages ranged from 5 to 12 years. All horses were deemed healthy based on normal physical exam, serum biochemistry and complete blood count findings prior to inclusion in the study. Horses were housed in box stalls beginning 24 h prior to drug administration and for the duration of the study. They were offered free choice water and hay with the exception that horses were fasted prior to misoprostol administration 10. To fast the horses, hay was removed 12 h prior to and 2 h after administration of misoprostol. Physical exams were conducted prior to drug administration, and 1, 2, 4, 6, 8, 10, 12 and 24 h following misoprostol administration. Parameters assessed included mentation, rectal temperature, heart and respiratory rates, capillary refill time, mucous membrane colour and hydration, digital pulse quality, intestinal borborygmi and number of faecal piles produced.

### Misoprostol administration

A single dose of misoprostol was administered in each phase of the study. Two hundred μg Cytotec [misoprostol] tablets^1^ at a dose of 5 μg/kg were dissolved in 30 mL of warm water in a 60‐mL catheter tip syringe for 5–10 min. Corn syrup (20 mL) was added immediately prior to administration to enhance palatability. Misoprostol suspensions were administered to horses in the left interdental space onto the back of the tongue, and the head was elevated for 30 s to ensure adequate drug delivery with minimal drug loss. The misoprostol dose (5 μg/kg) was chosen based on the dose routinely used in equine clinical settings 10, 20.

## Phase one methods: pharmacokinetic study

### Blood sampling

Blood sampling was accomplished by way of an indwelling catheter^2^ placed aseptically in the right jugular vein 1 day prior to drug administration. The total internal volume of the catheter plus extension tubing was 3.8 mL. Catheters (14 gauge, 5¼ inch) and extension tubing were sutured in place, and routine monitoring of the catheter and catheterisation site was performed throughout the study.

For sample collection, 10 mL of waste blood was collected through the catheter approximately 30 s prior to sampling and discarded to prevent dilution of the sample. Blood (7 mL) was then sampled through the catheter at time 0 (T0, prior to drug administration) as well as 10, 20, 30, 45, 60 and 90 min, and 2, 4, 6, 8, 12 and 24 h following oral misoprostol administration. Blood was immediately placed into lithium heparin vacutainer tubes. Catheters were flushed with 10 mL heparinised saline following each blood collection. Blood samples were immediately placed on ice for a maximum of 1 h prior to centrifugation at 2000 ***g*** for 10 min. Plasma was aspirated and stored in 2 mL cryogenic plastic tubes at −80°C until analysis.

### Chromatographic assay

Misoprostol is quickly metabolised in vivo to its active metabolite, misoprostol free acid, via rapid presystemic de‐esterification in the stomach to form a pharmacologically active carboxylic acid compound 21. Thus, misoprostol free acid (MFA) was measured in equine plasma as a reflection of misoprostol absorption. The concentrations of misoprostol free acid (MFA) were determined by ultra‐high pressure liquid chromatography with mass spectrometry. Calibration curves were prepared by fortifying blank equine plasma with stock solutions of misoprostol free acid^3^ and the internal standard, misoprostol free acid‐d5^3^, was dissolved in 100% methanol. Samples and standards were then prepared by adding 1 mL plasma to 1 mL of 1% formic acid in water in a glass tube, and vortexing for 15 s. The sample mixture was then added to supported liquid extraction cartridges (Isolute SLE + 2 mL)^4^ and a light vacuum was applied to initiate absorption. Two aliquots of 5 mL of methyl tert‐butyl ether were added to the cartridges, allowed to sit for 5 min and then slowly eluted under light vacuum. The resulting eluate was then placed in an evaporator and dried under a 20‐psi stream of nitrogen for 30 min at 44°C. Samples were reconstituted in 100 μL of 50:50 water:methanol (v/v). Volumes of 80 μL for samples and standards were injected on an Agilent Infinity 1290 system coupled with an Agilent G6530A Q‐TOF Mass Spectrometer^5^ run in ESI negative mode. Parameters for the ESI dual source were: drying gas, 9 L/min of nitrogen; nebulisation gas, 30 psi; sheath gas flow rate, 11 L/min; sheath gas temperature, 350°C; drying gas temperature, 350°C; capillary voltage, 4000 V; nozzle voltage, 1000 V; fragmentor voltage, 120 V; and CID collision gas, nitrogen. A gradient was used and the initial mobile phase was 5 mmol/L ammonium acetate buffer and 95:5 water:acetonitrile (50:50 v/v) for the first 10 min. The last 2 min of the run, the mobile phase was 5:95 (v/v). Flow rate was maintained at 0.5 mL/min. The selected ion recordings (SIR) used for misoprostol free acid and misoprostol free acid‐d5 were 367.25 and 372.29 respectively. Separation was achieved using an Agilent Zorbax SB C18 column (3.5 μm, 4.6 × 50 mm) and guard column^6^ maintained at 30°C. Under these conditions, the retention time for misoprostol free acid and misoprostol free acid‐d5 were 8.4 and 8.0 min respectively. Standard curves were linear over a concentration range of 0.05–50 ng/mL. The lower limit of quantification was 0.05 ng/mL, with an R^2^≥0.99.

### Data analysis

Pharmacokinetic analysis was performed using a one‐compartmental model using commercially available software (Phoenix WinNonLin, version 6.3.0)^7^ . The model was chosen based on best fit following visual analysis of the mean plasma concentration vs. time curve, as well as Akaike's information criteria. The maximal plasma concentration of MFA (C_max_) and the time at which MFA reached maximal concentration (T_max_) were derived directly from the data and are presented as mean ± s.d. Visual examination of the plasma concentration vs. time curves from preliminary analysis showed that one horse (horse A) had plasma concentrations of MFA that were considerably higher compared to the other horses. Data were therefore subjected to analysis using the Grubbs test to determine the presence of outliers.

Additional parameters reported include the total systemic drug exposure, as calculated by the area under the MFA serum concentration vs. time curve extrapolated to infinity (AUC_0−∞_), the first‐order absorption and elimination rate constants (k_01_ and k_10_, respectively), and the half‐life of absorption and elimination (k_01_ t_½_ and k_10_ t_½_, respectively) of misoprostol. As no i.v. administration of misoprostol was performed for comparison, bioavailability (F) of the drug could not be calculated. Thus, the primary pharmacokinetic parameters of volume of distribution (Vd) and clearance (Cl) are reported as Vd/F and Cl/F, which are dependent on bioavailability.

## Phase two methods: ex vivo inflammation model study

### Misoprostol administration and blood sampling

Thirty millilitres of heparinised whole blood were obtained via jugular venepuncture at T0, and 30 min, 1 and 4 h following oral misoprostol administration. Times were chosen based on the C_max_ and duration of drug persistence in the blood, as determined from phase one. Additionally, a blood sample was collected at each time point for measurement of MFA concentrations (per methods above), and an additional EDTA whole blood sample was also obtained at T0 and 30 min for complete blood count analysis (Table 1). Due to the decreased number of samples taken (total of four samples) and length of time of experimentation (4 h total) in this phase of the study, jugular catheters were not used to avoid potential complications of jugular catheter placement such as thrombophlebitis. Samples were taken from alternating sides, as well as alternating upper and lower quadrants of the vein to avoid excessive jugular trauma. Following collection, heparinised whole blood was aliquoted into sterile 15 mL polypropylene conical tubes^8^ and the erythrocytes were allowed to settle out of solution at room temperature for 1 h. The leucocyte‐rich plasma (LRP) layer was then collected and leucocytes were examined for viability via trypan blue exclusion. Leucocyte viability was routinely above 95% prior to and following oral misoprostol administration, and therefore all samples were included in the analysis.

**Table 1:** Effects of 5 μg/kg oral misoprostol on leucocyte counts before (0 h) and 0.5 h following administration

	Cell number (10^3^/μL)							
	Total WBC		Neutrophils		Monocytes		Lymphocytes	
Min post miso administration	0	30	0	30	0	30	0	30
Horse A	6.18	6.20	4.264	4.526	0.062	0.00	1.669	1.550
Horse B	6.72	6.00	2.755	3.00	0.067	0.18	3.696	2.160
Horse C	6.62	6.25	4.237	4.813	0.066	0.313	2.052	0.875
Horse D	6.72	6.46	5.174	4.845	0.067	0.065	1.142	1.292
Horse E	5.79	5.93	3.532	3.973	0.116	0.059	2.084	1.779
Horse F	5.54	5.65	3.158	2.543	0.166	0.113	2.161	2.656

Values were not significantly different before or after oral misoprostol administration (via paired *t* test or signed rank *t* test, where appropriate).

### Ex vivo LPS stimulation

One millilitre aliquots of LRP were placed into sterile polypropylene microcentrifuge tubes and treated with LPS (*E. coli* 055:B5)^9^ at 100 ng/mL final concentration, or the vehicle control for LPS (sterile PBS) for 2 h at 37°C. This concentration of LPS has been optimised previously in our laboratory for adequate stimulation of equine leucocyte TNFα mRNA production without affecting leucocyte viability in vitro 17.

### RNA isolation and first‐strand cDNA synthesis

RNA isolation and DNase materials were obtained from Qiagen^10^ . RNA was isolated using an RNEasy Mini Kit per manufacturer's instructions, with homogenation using a QIAshredder. One on‐column DNAse digestion was performed prior to RNA elution, as well as following RNA elution using RNase‐Free DNAse set. RNA was then cleaned up using an RNEasy mini kit per manufacturer's protocol. Final eluted RNA concentration was determined via a Nanodrop Spectrophotometer^8^. First‐strand cDNA synthesis was carried out with equal quantities of mRNA from each sample using the Superscript III Reverse Transcription System^8^ per manufacturer's instructions using random hexamers (50 ng/mL).

### Real‐time PCR

Real‐time PCR was performed using a Biorad MyIQ Single‐Color Real‐Time PCR Detection System^11^ . A PCR master mix was prepared for each sample, and each was dispensed in triplicate. Each well contained 1 × TaqMan Gene Expression Master Mix^8^, 1 × appropriate TaqMan primer and probe^8^, equal quantities of cDNA (ranging from 6 to 10 ng) and RNAse‐free water up to a final volume of 25 μL. No‐reverse‐transcriptase and no‐template controls were included to confirm the absence of genomic DNA and DNA contamination respectively. Equine‐specific TaqMan primers and probes were obtained from Invitrogen/Thermo Fischer's proprietary database of predesigned primers and probes^8^ (Assay ID for TNFα: Ec03467871, and β2M: Ec03468699). Invitrogen/Thermo Fischer validates these primers and provides the NCBI target sequence used to design the primer and probe, the 25‐base pair region of probe binding, and predicted size of the amplicon for each set 22, 23. In preliminary experiments, products of the PCR reaction were run on a 2% agarose gel and visualised using EZ Vision Three DNA Dye^12^ for product specificity. Amplification cycles were carried out per manufacturer's protocol as follows: 50°C for 2 min, one time; 95°C for 10 min, one time; 95°C for 15 s, followed by 60°C for 1 min (with enabled real‐time data collection), 40 times. The ΔΔCt method was used for data analysis using β2M as the housekeeping gene. We have previously identified β2M as a stably expressed housekeeping gene in equine leucocytes using the method developed by Radonic *et al*. 24 (data not shown).

### Statistical analysis

All mRNA data were analysed using SigmaPlot software,^13^ and are expressed as mean fold change ± s.e.m. and analysed using One Way RM ANOVA with post hoc Holm–Sidak multiple comparisons. Mean pulse, respiration rate, temperature, complete blood cell counts (10^3^ cells/μl) of total white blood cells (WBCs) and individual leucocyte populations were compared via paired *t* test.

## Results

### Misoprostol tolerance

In phase one of the study, horses showed few adverse effects, with the exception of one horse that displayed signs of abdominal discomfort (flank watching, laying down), soft manure and depressed mentation 30 min post dosing. This episode did not require veterinary intervention and resolved within 30 min of onset. One additional horse developed soft manure approximately 2 h post dosing that also resolved within 24 h. In phase two of the study, four of the six horses had at least one episode of soft manure over the 24‐h study period, but did not display any signs of abdominal discomfort. No differences were detected between physical exam parameters before dosing and at any time after misoprostol administration in either phase of the study (data not shown). Complete blood counts reflected no changes in white blood cell (WBC) number, or individual populations of neutrophils, monocytes or lymphocytes following misoprostol administration in phase two of the trial (Table 1).

### Misoprostol pharmacokinetics

**Figure 1:**
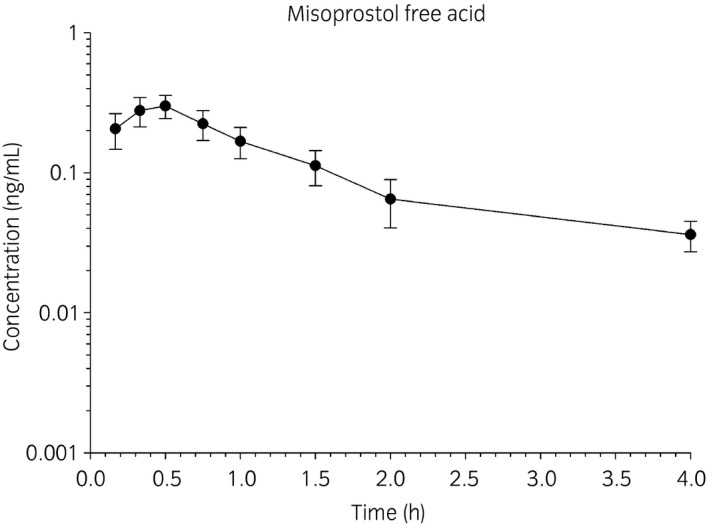
Mean plasma concentration of misoprostol free acid (ng/mL) vs. time (hours) after oral administration of misoprostol at a dose of 5 μg/kg. Data are represented as mean ± s.d. and include five different horses.

The mean plasma concentrations of MFA vs. time following oral administration of misoprostol to five horses are depicted in Figure 1. The C_max_ of horse A in phase I was 1.07 ng/mL at 30 min post misoprostol administration, compared to the mean C_max_ of 0.29 ng/mL of the other five horses. Statistical analysis confirmed that this horse was an outlier and therefore was excluded from analysis in this phase of the study (P<0.05). No additional outliers were identified within any other data sets. The MFA concentration at 30 min of horse A in phase II was only 0.19 ng/mL, therefore data from this horse was used in the analysis of the second phase. Misoprostol was absorbed quickly and resulted in detectable concentrations of MFA present in the plasma 15 min following oral drug administration in all horses. The T_max_ occurred at 23.4 ± 2.4 min following administration, and the C_max_ was 0.29 ± 0.07 ng/mL. Plasma MFA concentration increased with an absorption half‐life (k_01_ t_1/2_) of 7.2 ± 2.4 min, and declined with an elimination half‐life (k_10_ t_1/2_) of 40.2 ± 12 min. The mean total systemic exposure to the drug as extrapolated by AUC_0−∞_ was found to be 0.40 ± 0.12 ng h/mL (Table 2).

**Table 2:** Pharmacokinetics of oral administration of 5 μg/kg misoprostol in five horses

T_max_ (h)	0.39 ± 0.04	k_01_ t_1/2_ (h)	0.12 ± 0.04
C_max_ (ng/mL)	0.29 ± 0.07	k_10_ t_1/2_ (h)	0.67 ± 0.20
k_01_ (per h)	6.27 ± 1.57	AUC_0−∞_ (h ng/mL)	0.40 ± 0.12
k_10_ (per h)	1.10 ± 0.31	Vd/F	12.49 ± 2.38
		Cl/F	224.84 ± 59.89

Data represent five different horses. T_max_, time to maximum concentration; C_max_, maximum concentration; k_01_, first‐order absorption rate constant; k_10_, first‐order elimination rate constant; k_01_ t_1/2α_, half‐life of absorption; k_10_ t_1/2_, half‐life of elimination; AUC_0−∞_, area under the concentration–time curve extrapolated to infinity; Vd/F, apparent volume of distribution dependent on bioavailability; Cl/F, clearance dependent on bioavailability.

### The effect of misoprostol on ex vivo LPS‐induced leucocyte TNFα mRNA concentrations

**Figure 2:**
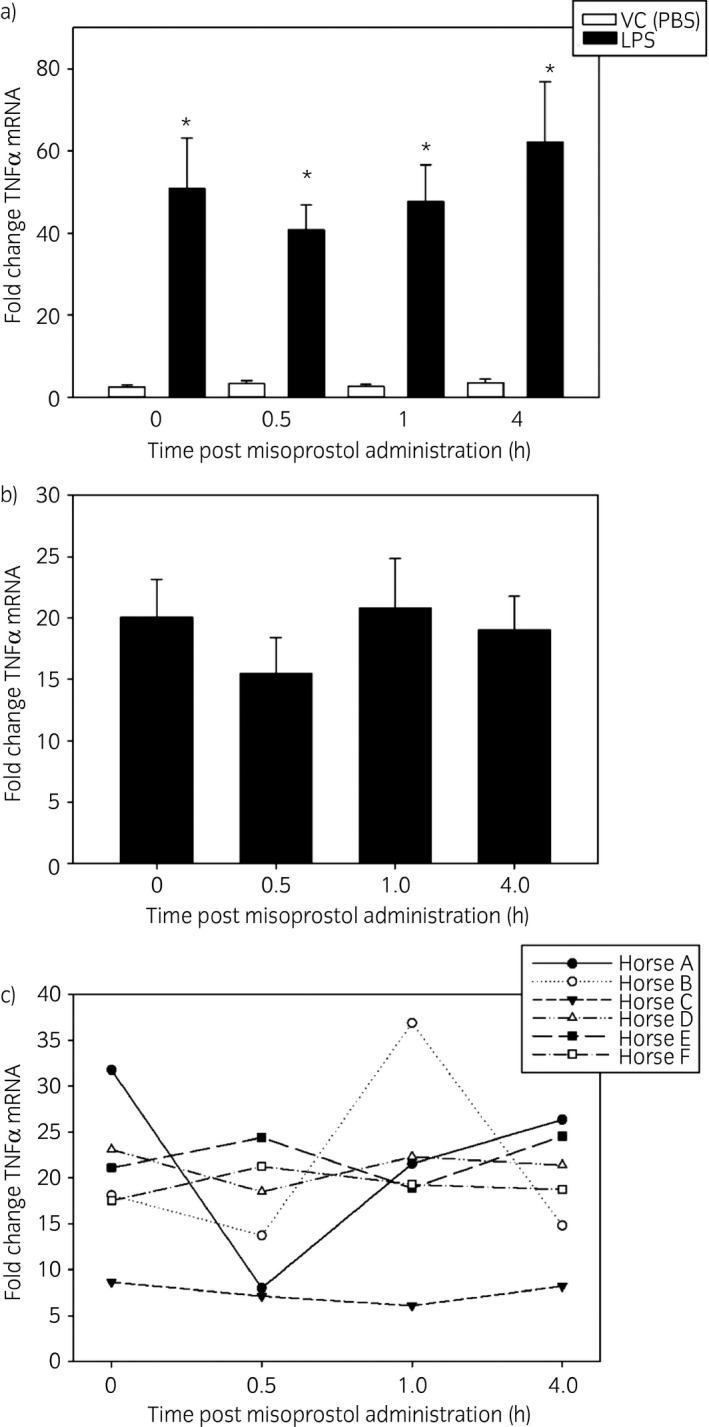
Effect of oral misoprostol administration on LPS‐stimulated equine leucocyte TNFα mRNA concentrations ex vivo. Following collection of whole blood (see methods section), one leucocyte‐rich plasma (LRP) sample per horse was immediately processed as a baseline measurement of TNFα mRNA concentrations. Remaining samples of LRP was incubated with either LPS (100 ng/mL) or vehicle (PBS) for 2 h. mRNA was isolated for real‐time PCR. a) LPS significantly increased TNFα mRNA concentrations at each time point evaluated. Data are presented as mean fold change TNFα mRNA ± s.e.m. vs. unstimulated baseline cells (not shown, equal to 1) and represent six horses. *P<0.05 vs. time‐matched vehicle treated cells (white bar) via paired *t* test. b) Misoprostol did not exert a statistically significant effect on LPS‐stimulated TNFα mRNA concentrations in equine leucocyte‐rich plasma. Data are presented as mean fold change TNFα mRNA ± s.e.m. vs. time‐matched vehicle‐treated unstimulated cells (not shown, equal to 1) and represent six different horses. c) Inter‐horse variability of the effect of misoprostol on LPS‐stimulated TNFα mRNA concentrations. Data are presented as in b).

No MFA concentration outliers were identified in phase two of the study, and therefore all six horses were included for analysis (Table 3). The mean plasma concentration of MFA at 30 min (approximate T_max_) in the second phase of the study was 0.25 ± 0.11 ng/mL, which is similar to the C_max_ found in phase one of the study (0.29 ± 0.07 ng/mL). Stimulation of equine leucocytes with 100 ng/mL of LPS led to an increase in TNFα mRNA concentrations compared with vehicle controls at all four time points sampled: prior to dosing (T0), at the approximate C_max_ (30 m), and 1 and 4 h after misoprostol administration (Fig 2a). Oral administration of misoprostol did not lead to a significant decrease in LPS‐stimulated TNFα mRNA production at any time point evaluated (P value 0.504, Fig 2b) High inter‐horse variability in TNFα mRNA production following oral misoprostol administration was noted (Fig 2c). Corresponding misoprostol free acid serum concentrations in each of the six horses in this phase of the study are reported in Table 3, and did not correlate with inter‐horse variability of TNFα mRNA inhibition.

**Table 3:** Plasma concentration of misoprostol free acid (ng/mL) in each horse in the second phase of study following oral administration of misoprostol at a dose of 5 μg/kg

Hours post miso administration	0	0.5	1	4
Horse A	Below LOQ	0.192	0.118	0.036
Horse B	Below LOQ	0.217	0.115	0.052
Horse C	Below LOQ	0.413	0.168	0.046
Horse D	Below LOQ	0.369	0.156	0.030
Horse E	Below LOQ	0.131	0.066	0.027
Horse F	Below LOQ	0.195	0.101	0.028
Mean	–	0.252	0.121	0.036
s.d.	–	0.111	0.037	0.010

## Discussion

These results demonstrate that misoprostol is absorbed following oral administration to horses and does not exert a significant effect on TNFα mRNA concentrations following LPS‐stimulation of equine leucocytes ex vivo. Additionally, this study is the first to define the pharmacokinetics of oral misoprostol administration in horses. Oral administration was chosen over i.v. administration due to documented adverse drug reactions, including acute collapse, in llamas following i.v. misoprostol dosing 25.

In this study, a single 5 μg/kg oral dose of misoprostol had a rapid absorption half‐life, short time to T_max_ and a rapid elimination half‐life in horses (Table 2). Misoprostol is a lipophilic methyl ester drug that is rapidly and extensively absorbed from the GI tract in humans 26, with relatively poor oral bioavailability when compared to other routes of administration such as sublingual or vaginal 27.While high lipophilicity promotes quick absorption, it is also associated with rapid metabolism in humans 28. In accordance with these properties, oral administration of misoprostol in men at doses (approximately 6–9 μg/kg) similar to those used in our study display a consistent mean T_max_ of 20–30 min and elimination half‐life of around 40 min 26, 27, 29, 30; these times are very similar to our findings in horses. Clinically, rapid absorption and elimination indicates that systemic effects of oral misoprostol will occur rapidly following administration, but will be relatively transient.

Reported human values for misoprostol C_max_ range from 0.28 to 2.05 ng/mL 27, 29, AUC_0−∞_ values range from 0.42 to 1.70 ng h/mL 29, 30 and a reported C_max_ CV between human subjects of 50% indicate that misoprostol absorption is highly variable 27. These wide‐ranging differences in concentration values were also observed in our equine study, and may be accounted for by well‐known concomitant parameters such as age, genetic differences and race/breed differences that affect absorption, elimination or metabolism of the drug between subjects. This variability could potentially be the cause of differences in anti‐inflammatory effects of misoprostol in horses within the same population. The plasma concentration of misoprostol which elicits anti‐inflammatory activity in vivo has not been determined. Human studies report a misoprostol IC_50_ of 750–955 nmol/L (approximately 287–365 ng/mL) for TNFα suppression in vitro in monocytes 31, 32. Additionally, in vitro equine studies demonstrate that misoprostol concentrations as low as 1 umol/L (approximately 383 ng/mL) significantly inhibit LPS‐induced TNFα mRNA production in equine leucocytes 17. While the mean C_max_ in this ex vivo study was orders of magnitude lower than seen in in vitro human and equine studies of TNFα inhibition, it is worth noting that in vitro data is likely not an accurate predictor of in vivo leucocyte responses to misoprostol. In vivo, leucocytes interact with many other cell types including vascular endothelial cells, which are critical to neutrophil and monocyte extravasation and migration to areas of tissue inflammation. Therefore, the ex vivo experimental model presented here may not entirely reflect the effects of misoprostol in vivo on these cell‐to‐cell interactions. In fact, in vivo mouse models demonstrate that misoprostol decreases neutrophil influx into tissues effected by cerebral haemorrhage 33. Misoprostol also provides tissue protective effects in rodent models of ischaemia‐reperfusion injury; these effects of misoprostol are mediated through interactions with EP2 and 4 receptors on endothelial cells 34, 35. Other anti‐inflammatory effects of misoprostol occur at much lower concentrations than those associated with TNFα production. In vitro equine studies report that misoprostol concentrations as low as 0.001 umol/L (approximately 0.38 ng/mL) inhibit LPS‐induced equine neutrophil respiratory burst 36, which is much more similar to the concentrations obtained in this study. Therefore, it is possible that misoprostol will affect leucocyte function directly and indirectly through its actions on other cell types in vivo.

While there is evidence of the anti‐inflammatory effects of misoprostol in in vitro cell models, use of misoprostol as an anti‐inflammatory in specific diseases warrants further study prior to application in clinical cases. For example, misoprostol has been shown to worsen T17‐cell mediated inflammation in rodent disease models of rheumatoid arthritis and inflammatory bowel disease 37, 38. Additionally, a novel EP4 receptor antagonist (piprant class, grapiprant) is currently marketed to decrease inflammation and pain associated with osteoarthritis in dogs 39; this novel compound was shown to decrease paw swelling, serum sialic acid concentrations, albumin/globulin ratios, synovial inflammation and bone destruction in rodent adjuvant‐induced arthritis models 40. Piprant compounds have yet to be studied in horses. These findings support the dynamic roles of EP receptors in different disease processes, and taken together, it is likely that targeting different combinations of EP receptors have different effects on inflammation depending on species, disease process and cell types involved. Therefore, use of misoprostol in various disease states in horses warrant further research.

In this study, a single oral dose of misoprostol did not significantly inhibit equine leucocyte TNFα mRNA production ex vivo. A lack of significant inhibition may be due to high inter‐horse variability of pharmacokinetic parameters, as has been observed in human species 27. Additionally, more potent stimulation of TNFα mRNA production occurred in this ex vivo model than has been reported in some equine in vivo experimental models; an approximately 50‐fold increase in TNFα mRNA production over baseline was reported in this study, which is greater than the approximately fivefold increase in TNFα mRNA production reported in in vivo equine models of endotoxaemia 41. Furthermore, low MFA C_max_ produced in this model may also cause the lack of statistically significant inhibition of TNFα mRNA production. A recently published human trial demonstrated a significant decrease (29%) in TNFα concentrations following oral misoprostol administration using an ex vivo inflammation model similar to our design 42. This human trial differed from our study in that they used a repeat dosing strategy of 100–300 μg misoprostol four times daily for 14 days. While clinical dosing of misoprostol in horses uses a q6–12 h‐dosing interval, it is unlikely that a repeat dosing regimen would achieve higher MFA C_max_ values in horses due to the short half‐life and lack of persistence of MFA in the plasma. However, one study has demonstrated that while peak MFA plasma concentrations remained relatively stable after q3h misoprostol administration in human subjects, the AUC and therefore total systemic drug exposure was increased following repeat dosing 43. Therefore, repeat misoprostol administration could exert a cumulative effect on cells over longer periods, and has yet to be explored in horses.

An alternative method of increasing peak plasma concentrations of misoprostol is higher dosing. However, this is likely to result in gastrointestinal disturbance. Administering over 400–800 μg misoprostol per day in human subjects is associated with diarrhoea, nausea and abdominal cramping 26. The dose used in this study (5 μg/kg) is routinely and safely employed in horses clinically 10, 18, 20, but it is unknown if higher doses of misoprostol would cause episodes of colic. In horses, i.v. PGE_1_ administration at doses of 25–75 ng/kg/min over 120 min exerted dose‐dependent reductions in gastric, jejunual and colonic motility, as well as decreased borborygmi and mild abdominal discomfort 44. In accordance with these findings, one horse in the first phase of our study developed signs of colic within 30 min of dosing, as evidenced by soft faeces, laying down, flank watching and a dull mentation. Additionally, four of the six horses in the second phase of this study developed one to two episodes of soft faeces. While no medical intervention was needed, it is possible that increasing the dose of misoprostol could potentially lead to more severe effects as described previously 44. In human medicine, misoprostol is also used as an abortifacient 45, although a single study assessing the safety of misoprostol in mid‐term pregnant mares at 5 μg/kg orally, twice daily showed no adverse effects over a 5‐day course of treatment 19.

The results of this study do not provide enough information to justify alterations to the current commonly used dosing regimens of misoprostol. The short half‐life of misoprostol in the horse suggests that accumulation is unlikely to occur unless doses are greatly increased or dosing intervals are greatly shortened. This may lead to an increased risk of adverse effects and would require further study before use in clinical cases. For treatment of localised lesions, increased systemic exposure may not be necessary. A dose of 5 μg/kg administered orally twice daily has been shown to be effective for the treatment of equine gastric glandular disease (EGGD) 18, suggesting that it is possible misoprostol effectively inhibits inflammation locally within the stomach.

The horses in this study were fasted for 12 h prior to misoprostol administration and 2 h following administration to normalise the gastric environment across horses and to minimise barriers to misoprostol absorption. Fasting of horses has been performed previously in other studies utilising oral misoprostol dosing 10; however, as misoprostol pharmacokinetics were not evaluated in that study, this was a novel study design. It is possible that feeding horses prior to oral administration could affect the overall misoprostol absorption, however, the effect of food on misoprostol absorption remains to be elucidated.

In lieu of altered dosing strategies, another potential method of increasing the anti‐inflammatory efficacy of misoprostol is co‐administration with other anti‐inflammatory medications, which has been studied in vitro. Previous studies in human cells suggest that misoprostol could be used to enhance the anti‐inflammatory effects of the NSAIDs piroxicam, indometacin, and sodium salicylate on human neutrophil ROS production, degranulation and aggregation in vitro 46. Additionally, misoprostol augments the anti‐inflammatory effects of the COX‐2‐selective drug etoricoxib in vivo in rodent models of carrageenan‐induced paw inflammation 47. Thus, addition of misoprostol to existing NSAID therapy regimens could potentially augment the anti‐inflammatory effects of NSAIDs without increasing the risk of adverse effects; however, this requires further investigation in the horse before it can be recommended.

While this study failed to show a significant effect of oral misoprostol on equine leucocyte TNFα mRNA production ex vivo, it is possible that other agents that increase cAMP or other EP2 and EP4 agonists may have a more significant anti‐inflammatory effect. For example, pentoxifylline (a phosphodiesterase inhibitor that increases intracellular cAMP) decreases severity of equine models of severe equine asthma 48 and produces mild beneficial effects in experimental equine endotoxaemia models by decreasing rectal temperature, respiratory rate and prolonging time to re‐calcification of whole blood 49. Taken with our study, oral agents that increase cAMP have the potential to decrease inflammation in equine disease.

In conclusion, this study is the first to determine the pharmacokinetics of oral misoprostol in horses at a dose has been anecdotally used in horses, 5 μg/kg. This data reveals that oral misoprostol rapidly reaches peak plasma concentration, and this C_max_ is lower than values previously shown to elicit anti‐inflammatory effects on leucocytes in vitro. In accordance with these findings, oral misoprostol did not exert a significant effect on equine leucocyte TNFα mRNA production ex vivo. However, a limitation of this study was use of a single dosing strategy and a small group of horses. Therefore, different dosing strategies including repeat dosing or pairing with other anti‐inflammatory drugs in a larger group of animals warrants further study. Additionally, other cAMP‐elevating agents may produce more leucocyte‐modulating effects in vivo and have yet to be tested.

## Authors’ declaration of interests

No competing interests have been declared.

## Ethical animal research

All experiments were approved by the Institutional Animal Care and Use Committee at North Carolina State University.

## Sources of funding

Morris Animal Foundation, Grant # D15EQ018.

## Authorship

E.M. Martin was responsible for study design, experimental execution, data analysis and interpretation and preparing the manuscript. J.M. Schrimer was responsible for technical support and manuscript preparation and review. S.L. Jones was responsible for study design and critically reviewing the manuscript. J.L. Davis was responsible for overseeing all aspects of the study including study design, experimental execution, data analysis and interpretation and critically reviewing the manuscript.

## Manufacturers' addresses

1 Gavis Pharmaceuticals, Somerset, New Jersey, USA.

2 Mila International, Inc., Florence, Kentucky, USA.

3 Cayman Chemical, Ann Arbour, Michigan, USA.

4 Biotage, Charlotte, North Carolina, USA.

5 Agilent Technologies, Santa Clara, California, USA.

6 Waters Corporation, Milford, Massachusetts, USA.

7 Pharsight Corporation, Cary, North Carolina, USA.

8 Thermo Fischer Scientific, Grand Island, New York, USA.

9 Sigma‐Aldrich, St. Louis, Missouri, USA.

10 Qiagen, Valencia, California, USA.

11 Biorad, Hercules, California, USA.

12 Amresco, Solon, Ohio, USA.

13 Systat Software, San Jose, California, USA.
